# Association between Smoking and Latent Tuberculosis in the U.S. Population: An Analysis of the National Health and Nutrition Examination Survey

**DOI:** 10.1371/journal.pone.0049050

**Published:** 2012-11-08

**Authors:** David J Horne, Monica Campo, Justin R. Ortiz, Eyal Oren, Matthew Arentz, Kristina Crothers, Masahiro Narita

**Affiliations:** 1 Division of Pulmonary and Critical Care Medicine, Department of Medicine, University of Washington, Seattle, Washington, United States of America; 2 Department of Global Health, University of Washington, Seattle, Washington, United States of America; 3 Public Health - Seattle & King County, TB Control Program, Seattle, Washington, United States of America; 4 Department of Epidemiology, University of Washington, Seattle, Washington, United States of America; San Francisco General Hospital, University of California San Francisco, United States of America

## Abstract

**Background:**

Evidence of an association between cigarette smoking and latent tuberculosis infection (LTBI) is based on studies in special populations and/or from high prevalence settings. We sought to evaluate the association between LTBI and smoking in a low prevalence TB setting using population-based data from the National Health and Nutrition Examination Survey (NHANES).

**Methods:**

In 1999–2000, NHANES assessed LTBI (defined as a tuberculin skin test measurement ≥10 mm) in participants, and those ≥20 years of age were queried regarding their tobacco use and serum cotinine was measured. We evaluated the association of LTBI with self-reported smoking history and smoking intensity in multivariable logistic regression models that adjusted for known confounders (gender, age, birthplace, race/ethnicity, poverty, education, history of BCG vaccination, and history of household exposure to tuberculosis disease).

**Results:**

Estimated LTBI prevalence was 5.3% among those ≥20 years of age. The LTBI prevalence among never smokers, current smokers, and former smokers was 4.1%, 6.6%, and 6.2%, respectively. In a multivariable model, current smoking was associated with LTBI (OR 1.8; 95% CI, 1.1–2.9). The association between smoking and LTBI was strongest for Mexican-American and black individuals. In multivariate analysis stratified by race/ethnicity, cigarette packs per day among Mexican-American smokers and cotinine levels among black smokers, were significantly associated with LTBI.

**Conclusions:**

In the large, representative, population-based NHANES sample, smoking was independently associated with significantly increased risks of LTBI. In certain populations, a greater risk of LTBI corresponded with increased smoking exposure.

## Introduction

One-third of the world’s population is estimated to have latent tuberculosis infection (LTBI) and in 2010, 8.8 million people had tuberculosis disease (TB) [1]. While it has been almost 2 decades since the World Health Organization (WHO) declared tuberculosis a global emergency [2], TB remains a leading cause of death worldwide [1]. As a TB control strategy, WHO and other public health organizations recognize the importance of prevention of the most frequent TB risk factors [3]. In 2007, several systematic reviews identified an association between smoking and TB, calling attention to the potential role of smoking in TB pathogenesis and its contribution to global disease burden [4]–[7]. For these reasons, WHO recommends tobacco cessation be part of TB control programs [3].

The relationship between tobacco and LTBI is less clear. LTBI is an asymptomatic state that is diagnosed by evaluation of the host immune response through tuberculin skin tests (TST) or interferon-gamma release assays [8]. In addition to assessing the relationship with TB disease, the 2007 systematic reviews evaluated the evidence for an association between smoking and LTBI [4]–[6]. The included studies were limited to special populations, including prisoners, migrant workers, immigrants, and the homeless [9]–[13], aside from one population-based study that was from a very high TB prevalence setting in South Africa [14]. These studies were limited by potential misclassification of smoking behavior and variation in adjustment for potential confounders [4]. Although meta-analysis of the studies showed an overall association between smoking and LTBI, the evidence was weaker than for TB disease [15]. Furthermore, a recent study from South Africa found no association between smoking and LTBI [16].

There are no population-based studies from areas of low TB prevalence that evaluate the association between smoking and LTBI [4]. Prior studies on associations between smoking and LTBI are not generalizable to low TB incidence countries [9]–[13]. In the US, a better understanding of associations between cigarette smoking and LTBI could improve the identification of at risk populations for LTBI screening.

The National Health and Nutrition Examination Survey (NHANES) is designed to assess the health and nutritional status of U.S. residents. NHANES 1999–2000 included an assessment of LTBI status, TB risk factors, smoking history, and serum cotinine levels [17], [18]. We evaluated associations between self-reported smoking status and LTBI after adjusting for confounders. We also assessed associations between LTBI and intensity of smoking as defined by daily pack intensity, overall exposure history, and serum cotinine levels. Finally, we compared the results of self-reported smoking history to measured serum cotinine levels.

## Materials and Methods

### Study Population

NHANES is a continuous program of studies conducted by the U.S. Centers for Disease Control and Prevention to assess the health and nutritional status of residents [19]. NHANES subject selection is designed to obtain a representative sample of the U.S. civilian non-institutionalized population. NHANES 1999–2000 collected data on participant demographics, health history, tobacco history, and tuberculosis risk factors [20]. We included participants 20 years of age and older as younger participants were administered a different tobacco history questionnaire.

### Data Collection

NHANES offered participants skin tests with a tuberculin-purified protein derivative (PPD) product, PPD S-1 [21]. Trained phlebotomists injected 0.1 ml of PPD intradermally using the Mantoux method and trained TST readers measured reactions 48–72 hours later. Participants with a history of a severe reaction to a prior TST were excluded. NHANES defined LTBI as an area of induration ≥10 mm in response to PPD regardless of participants’ TB risk factors [17], [18].

NHANES ascertained smoking history with the following question: “Have you smoked at least 100 cigarettes in your entire life?” Participants who responded affirmatively were considered ever smokers and were queried on current smoking status, “Do you now smoke cigarettes?” A response of “Every day” or “Some days” was coded as current smoker while a response of “Not at all” was coded as former smoker. Current and former smokers were asked about daily cigarettes smoked currently and at the time of quitting, respectively. We estimated packs per day as the self-reported number of cigarettes smoked per day divided by twenty and rounded to the nearest integer; and we assigned a value of one pack per day to those who smoke 1 to 20 cigarettes daily. We defined pack-years of cigarettes as the number of cigarettes per day smoked multiplied by years of smoking, divided by twenty, and rounded to the nearest integer.

We included known and potential risk factors for LTBI and TST reactivity in multivariable analyses: age, gender, race/ethnicity, poverty, education, exposure to household contacts with active tuberculosis, foreign birth, and prior bacillus Calmette-Guerin (BCG) vaccination [17], [18]. In order to reduce the risk of disclosure, NHANES assigned adults ages 85 years and older an age of 85 years. NHANES defined race/ethnicity based on self-report and categorized respondents as white (non-Hispanic), black (non-Hispanic), Mexican-American, other Hispanic, and other; due to small number of participants in the latter two groups these were combined in our study [22]. Poverty was defined as a poverty/income ratio < one; the poverty/income ratio is the family income over the prior 12 months divided by the poverty threshold and is adjusted for family size [17]. Education was categorized as less than high school, high school graduate or equivalent, and education beyond high school. To evaluate a known history of tuberculosis exposure, subjects were asked “Have you ever lived in the same household with someone while that person was sick with tuberculosis or TB?” BCG vaccination leaves an identifiable scar in most individuals [23], and past BCG receipt was assessed through visual inspection by NHANES staff.

Cotinine is a major metabolite of nicotine that may be used as a marker for active smoking. Serum cotinine was measured by isotope dilution-high performance liquid chromatography/atmospheric pressure chemical ionization tandem mass spectrometry; cotinine concentrations were derived from the ratio of native to labeled cotinine in the sample by comparisons to a standard curve [24]. The laboratory limit for detection of cotinine was 0.05 ng/mL [25]. We used cotinine levels to address two issues in these analyses. First, we used cotinine levels to estimate the accuracy of self-reported current smoking status using a cotinine level of 3 ng/mL to define active smoking [25]. Second, we used cotinine levels to measure the intensity of cigarette exposure and model an association with LTBI.

### Statistical Analysis

For all questions, we coded a refusal to answer or response of “don’t know” as missing. Bivariate analyses were performed using the chi-square test for categorical variables or t-test for means when appropriate. All tests were two-sided and the level for determining statistical significance was set at p≤0.05. We accounted for the survey design by using the Taylor series linearization method to estimate variances and sampling errors [22]. We calculated prevalence estimates using the NHANES Mobile Examination Center (1999–2000) 2- year weights and calculated additional weights based on TST nonparticipation to correct for bias in our regression analyses [22], [26]. All of our pre-selected variables were included in the final multivariable models based on our initial hypotheses; subjects with missing data for variables of interest were excluded from regression analyses. There were low levels (<0.3%) of missing data among the variables that selected for inclusion in our models with the exception of poverty (13.3%). High levels of missing data were present for HIV status (49.7%); we did not include HIV status as a variable in the regression models due to the previously described absence of HIV-seropositive subjects with ≥5 mm of induration on TST testing in this NHANES survey [18]. In this cross-sectional study, we used logistic regression models to analyze associations between LTBI and self-reported history of current and former smoking compared to life-long non-smokers in the entire sample and stratified by race/ethnicity. We also assessed associations between LTBI and smoking intensity as defined by self-reported packs per day, pack-years of smoking, and measured serum cotinine levels. Data analyses were performed using Stata 11 (StataCorp, College Station, TX).

We performed several sensitivity analyses. We restricted the multivariate analysis of smoking status and LTBI to participants whose serum cotinine levels were concordant with self-reported smoking status (i.e. current smokers with serum cotinine ≥3 ng/mL and never/former smokers with levels <3 ng/mL). Heavy alcohol use has been reported to affect the strength of the relationship between cigarette smoking and LTBI [5]. Due to high proportions of missing data for alcohol use (19%), we evaluated the effect of heavy drinking (≥5 more drinks per day) in sensitivity analyses.

## Results

### Baseline Characteristics

A total of 4880 participants in NHANES 1999–2000 were 20 years of age or older, of whom 3843 (78.8%) had TST results. LTBI was detected in 339 of the study participants (8.8% of those with TST results). In a weighted analysis, we estimated LTBI prevalence in the U.S. among individuals ≥20 years of age as 5.3% in 1999–2000. Estimates of LTBI prevalence among sub-groups are presented in Table 1. Current smokers had a significantly higher prevalence of LTBI compared to never smokers. Consistent with previously reported estimates, a higher prevalence of LTBI was observed among a number of subgroups including men, foreign-born, non-whites, the poor, less educated, BCG vaccinated, and those with a history of TB exposure [18].

**Table 1:** Estimated latent tuberculosis infection prevalence by characteristics in the U.S. population age ≥20 years.

Characteristic	Number Tested	Unadjusted Prevalence (%) with LTBI (95% CI)
All subjects	3829	5.27 (4.07–6.47)
Smoking		
Never smoker	2000	4.09 (2.37–5.80)
Current smoker	795	6.56 (4.41–8.71)
Former smoker	1028	6.20 (3.96–8.44)
Age group (years)		
20–39	1341	4.41 (2.39–6.44)
40–59	1100	6.15 (4.34–7.95)
60+	1388	5.60 (4.02–7.18)
Gender		
Women	2033	3.87 (2.41–5.32)
Men	1796	6.81 (5.44–8.17)
Birth place		
U.S. born	2835	2.39 (1.69–3.10)
Foreign born	994	20.25 (14.03–26.47)
HIV seroprevalence		
HIV seronegative	1917	4.71 (3.27–6.14)
HIV seropositive	15	0
Race/ethnicity		
White	1768	2.23 (1.23–3.22)
Black	723	10.38 (7.66–13.10)
Mexican-American	1029	13.93 (10.99–16.88)
Other	309	15.23 (9.05–21.42)
Poverty		
Poverty income index >1	2649	4.23 (3.00–5.47)
Poverty income index <1	675	8.24 (4.68–11.81)
Education		
Less than high school	1483	10.36 (7.95–12.78)
High school or equivalent	873	3.52 (2.17–4.88)
More than high school	11473	3.65 (2.10–5.21)
History of heavy alcohol use		
No	2529	4.52 (3.24–5.80)
Yes	572	6.25 (4.40–8.10)
History of TB exposure	183	15.88 (8.01–23.74)
No history of TB exposure	3626	4.84 (3.62–6.07)
History of BCG vaccination		
No	3295	4.31 (3.15–5.47)
Yes	534	11.57 (5.91–17.22)

Definition of abbreviations: CI - confidence interval; LTBI - latent tuberculosis infection.

### Smoking Related Characteristics

A history of smoking as defined by more than 100 lifetime cigarettes was present in 49.7% of the population (95% confidence interval [CI], 46.2–53.3), of whom 49.3% (45.3–53.4) were former smokers and 50.6% (46.6–54.7) were current smokers. On average, smoking started at age 17.9 years (16.7–19.1) in all smokers and age 18.4 years (16.4–20.4) in foreign born smokers. Former smokers reported higher packs per day smoked at the time of quitting (1.29 packs per day, 1.24–1.35) and more pack-years (21.03, 19.1–22.9) than current smokers (1.23, 1.18–1.28, and 13.0, 11.3–14.7, respectively). Among never smokers and former smokers, cotinine levels averaged 8.2 ng/mL (5.5–10.9) and 22.8 ng/mL (11.0–34.5), respectively, compared to 196.6 ng/mL (186.9–206.4) in current smokers. Using serum cotinine levels to verify self-reported smoking status, serum cotinine levels were 3 ng/mL or higher in 96.2%, 11.8% and 5.2% of current, former, and never smokers, respectively. There were differences in smoking status by race/ethnicity (Table 2). Regardless of smoking status, black persons had higher serum cotinine levels compared to other races/ethnicities.

**Table 2:** 2010 NHANES participant smoking-related characteristics by race/ethnicity.

	Race/ethnicity			
Characteristic	White	Black	Mexican-American	Other
Smoking status (%)				
Never smoker	47.1 (43.2–50.9)	58.0 (51.1–64.8)	59.4 (53.6–65.1)	58.6 (48.9–68.3)
Current smoking	25.4 (21.6–29.2)	27.2 (22.5–32.0)	19.8 (15.0–24.6)	24.6 (15.4–33.8)
Former smoking	27.5 (24.7–30.3)	14.8 (11.2–18.3)	20.8 (17.9–23.7)	16.7 (10.1–23.5)
Pack-years				
Current smoking	15 (13–17)	8 (6–10)	6 (5–8)	7 (5–10)
Former smoking	22 (20–25)	19 (12–26)	10 (7–14)	15 (8–22)
Packs per day				
Current smoking	1.3 (1.2–1.3)	1.0 (1.0–1.1)	1.0 (1.0–1.1)	1.0 (1.0–1.1)
Former smoking	1.3 (1.2–1.4)	1.2 (1.1–1.3)	1.1 (1.1–1.2)	1.2 (1.0–1.3)
Cotinine level, ng/mL				
Never smoker	9 (4–13)	18 (5–30)	2 (0–3)	1 (0–2)
Current smoking	204 (193–216)	243 (225–261)	121 (92–150)	133 (108–159)
Former smoking	23 (9–37)	39 (30–49)	5 (1–9)	21 (5–38)

Numbers in parentheses refer to 95% confidence intervals.

### Smoking Status and LTBI Prevalence

In bivariate analyses, current smokers (odds ratio [OR] 1.65, 95% CI 1.11–2.46), but not former smokers (OR 1.55, 0.80–3.00), were at increased risk for LTBI. This finding was confirmed in a multivariable logistic regression model adjusting for known LTBI risk factors, where we observed a significant independent association between LTBI and current smoking (OR 1.72, 1.04–2.86), but not former smoking (OR 1.87, 0.90–3.91) (Table 3).

**Table 3:** Multivariable model of smoking status and LTBI in all subjects.

Factor	Odds Ratio	95% Confidence Interval
Smoking history		
Non-smoker	referent	
Former smoker	1.76	0.82–3.75
Current smoker	1.76	1.06–2.94
Age (years)	1.02	1.01–1.03
Sex		
Female	referent	
Male	1.78	1.19–2.66
Birthplace		
U.S.	referent	
Foreign	6.23	3.60–10.78
Poverty		
Poverty income index <1	referent	
Poverty income index >1	1.32	0.81–2.16
History of TB exposure	4.41	1.87–10.37
BCG vaccination	1.46	0.84–2.52
Education		
Less than high school	referent	
High School	0.77	0.44–1.35
More than high school	0.88	0.52–1.50
Race/ethnicity		
White	referent	
Black	5.26	2.71–10.21
Mexican-American	2.89	1.19–7.05
Other race/ethnicity	2.96	1.18–7.39

### Smoking Status and LTBI Prevalence within Race/ethnicity Subgroups

We observed differences in the strength of the associations between smoking status (current and former smoking) and LTBI when using weighted versus unweighted (not presented) models. This suggests that associations between smoking status and LTBI differ by racial/ethnic subgroups that are over represented in the sample compared to population demographics. Therefore, we analyzed the association between LTBI and smoking status stratified by race/ethnicity in multivariable models that adjusted for age, gender, birth place, poverty/income ratio, history of TB exposure, history of BCG vaccination and education level (Table 4). Current smoking was significantly associated with LTBI among black persons (OR 1.90, 1.03–3.50) and Mexican-American (OR 2.12, 1.55–2.89) populations, but not among white or other populations. Among Mexican-American persons, former smoking was also associated with LTBI (OR 3.12, 1.33–7.31).

**Table 4:** Multivariable logistic regression of LTBI in analyses stratified by race/ethnicity.

Race/Ethnicity	OR (95% CI) for LTBI	
	Current smoker	Former Smoker
White	1.47 (0.53–4.09)	2.38 (0.85–6.66)
Black	1.90 (1.03–3.50)	0.79 (0.31–2.01)
Mexican American	2.12 (1.55–2.89)	3.12 (1.33–7.31)
Other	1.66 (0.80–3.45)	1.37 (0.64–2.90)

Each race/ethnicity modeled separately and adjusted for age, gender, birth place, poverty:income ratio, history of TB exposure, history of BCG vaccination and education level.

### Intensity of Smoking Exposure and LTBI

We next evaluated the association between exposure to cigarettes and LTBI, modeling the degree of exposure to cigarettes in three different ways: self-reported packs per day, to reflect daily intensity presently or at the time of quitting, self-reported pack-years to measure intensity and duration of exposure, and serum cotinine levels, hypothesized to reflect a subject’s level of smoking intensity. Smoking status was not included in these models. In multivariate analyses of the entire cohort, neither packs per day nor pack-years were associated with LTBI (OR 1.30, 0.97–1. 67 and 1.00, 0.99–1.01, respectively). In analyses stratified by race/ethnicity, a significant association was observed among Mexican-Americans, OR 2.52 for each additional pack per day smoked (1.60–3.99), but not in other races/ethnicities. We did not find an association between total pack-years and LTBI among all subjects with a history of smoking, or when stratified by race/ethnicity.

Modeled as a continuous variable, cotinine levels were not independently associated with increased risk for LTBI in the sample as a whole. However, when analyzed by race/ethnicity, higher cotinine levels were associated with an increased risk of LTBI in black subjects: every 100 ng/mL increase in cotinine was associated with an OR of 1.28 for LTBI (1.06–1.55); this association was observed only among current black smokers.

### Sensitivity Analyses

When multivariable models were restricted to subjects whose self-reported smoking status was concordant with measured serum cotinine levels (2928 observations), we observed significant associations between LTBI and smoking status similar to those previously noted among all current smokers (OR 1.85, 1.04–3.29), black current smokers (OR 2.20, 1.01–4.79), and Mexican-American current (OR 2.48, 1.75–3.52) and former smokers (OR 3.03, 1.18–7.78).

We evaluated whether the inclusion of heavy alcohol use confounded the observed associations between smoking status and LTBI. In a multivariable model that contained all subjects with data on alcohol use (n = 2695), there was no association between LTBI and heavy alcohol use (OR 0.81, 95% CI 0.398–1.66). However, the inclusion of heavy alcohol use strengthened the associations of current and former smoking with LTBI (OR 2.47, 1.63–3.76, and 2.57, 1.21–5.49, respectively).

## Discussion

We evaluated associations between cigarette smoking and LTBI in a large representative sample of the U.S. population and found an independent association between current cigarette smoking and LTBI. While meta-analyses have found a significant relationship between smoking and LTBI [4]–[6], the included studies were conducted in special populations at high risk for LTBI (i.e. homeless, incarcerated, and Vietnamese immigrants). Our population-based findings remained robust to sensitivity analyses that included serum cotinine verification of self-reported smoking status. Our findings suggest that in low TB prevalence countries cigarette smoking is a strong risk factor for LTBI.

In the analyses stratified by race/ethnicity, we observed a significant risk for LTBI in black and Mexican-American current smokers, in Mexican-American former smokers, and in Mexican-Americans and black persons by different measures of smoking intensity. There are several possible explanations for differences in the observed associations in risk of LTBI by race/ethnicity. There may be a true difference in how race/ethnicity affects the association between smoking and LTBI that could be due to innate or acquired characteristics. Differences by ethnicity have been observed in the metabolism of nicotine and other products of cigarettes, and levels of nicotine intake per cigarette in black individuals [27]; we found that compared by smoking status black participants had higher serum cotinine levels compared to other races/ethnicities. There is a lack of data on whether race/ethnicity modifies associations between LTBI and tobacco as all studies of associations between LTBI and cigarette smoking have included subjects from non-white backgrounds [10]–[12], [14], [16], with the exception of one study from the United States in which 76% of participants were black [9], and one study conducted among Spanish participants that did not describe the race/ethnicity of participants [13]. Alternatively, it is possible that cigarette smoking affects TST results differently by race/ethnicity. Finally, there may be unmeasured confounders, particularly related to socioeconomic status.

To our knowledge, only one study has shown a significant independent association between smoking exposure and LTBI [12]. In this study of Vietnamese immigrants to Australia, duration of smoking in years was associated with LTBI (OR 1.04, 1.01–1.06). We used several metrics to assess smoking intensity in current and former smokers. Although we did not detect a difference in measures of smoking intensity and LTBI in the entire population, when we analyzed by race/ethnicity we found reported packs per day associated with LTBI in Mexican-American current and former smokers, and cotinine levels associated with LTBI in current black smokers. These findings support a dose-response relationship between smoking and LTBI. There is biological data to support a causal relationship between smoking and LTBI [28], [29]. Alveolar macrophage may have a role key in determining whether an individual develops LTBI following exposure to *M. tuberculosis* [30]. Macrophages from smokers may be less successful in controlling intracellular *M. tuberculosis* compared to never smokers [28].

There are limitations to our study. NHANES is a cross-sectional study and we were unable to determine if the initiation of cigarette smoking preceded the tuberculosis contact that led to LTBI. Among all study participants the mean age of smoking initiation was 17.9 years and in the foreign born 18.4 years. As the mean age of immigration in foreign-born participants was 37.7 years (35.0–40.4), it is likely that exposure to TB occurred while an individual was an active smoker. Second, our definition of LTBI was based on the TST which may produce both false positive and false negative results [25]. However, we believe that the TST limitations were minimized by adjusting for a history of BCG vaccination, and our conforming to a definition for LTBI that has been previously used in NHANES 1999–2000 [17], [18]. Third, misclassification of smoking status may have occurred; however, we verified self-reported status in current smokers with serum cotinine levels. Finally, there may be residual confounding.

In 2007, WHO published a monograph on integration of tobacco control into TB program activities [3]. However, there are no recommendations regarding smoking and LTBI screening. Smoking status may identify individuals with higher prevalence of LTBI and increased risk for progression to active tuberculosis [4], [5]. Associations between smoking, LTBI, and TB disease support consideration of LTBI screening in smokers from high-risk populations that would not be screened based on current guidelines. This screening could also provide an opportunity for smoking cessation interventions among active smokers, particularly among those diagnosed with LTBI [31].

In conclusion, the present study provides significant evidence of the independent relationship between cigarette smoking and LTBI. Our population-based study from the United States has important implications for other low TB prevalence settings in addressing TB control and elimination strategies.
